# Corporate communication as discursive decriminalization and symbolic compliance in transnational corruption criminal proceedings

**DOI:** 10.1007/s10611-026-10290-5

**Published:** 2026-06-11

**Authors:** Franziska Hohl Zürcher, Sophie Serrano

**Affiliations:** Center for Criminological Research, University of Neuchâtel, Neuchâtel, Switzerland

**Keywords:** Corporate corruption, Corporate communication, Offender, Discourse analysis, Narratives, Framing, Decriminalization, Symbolic compliance, Criminal proceedings

## Abstract

This study examines how multinational corporations strategically communicate their own corruption during criminal proceedings. Through qualitative content analysis, we analyze press releases and annual reports from 10 corporations convicted in Switzerland for organizational deficiencies in relation to bribery of foreign public officials between 2011 and 2023. Drawing on corporate decriminalization and symbolic compliance theory, we investigate corporate communication’s timing, framing strategies, and sequencing before, during, and after their criminal proceedings. Our results show that corporate communication is primarily reactive, driven by market regulations, media or public scrutiny, and judicial authority disclosures. We identify four distinct framing strategies employed by corporations to address their corruption cases: criminal frames acknowledging legal gravity while emphasizing compliance or cooperation, illegal frames downplaying severity by avoiding criminal terminology, managerial frames recasting corruption as technical mistakes, and righteous frames denying wrongdoing entirely. Companies initially minimize or deny corruption allegations, then acknowledge criminality when authorities announce conclusions from proceedings, before gradually returning to downplaying strategies. This dynamic framing serves reputation management while responding to stakeholder expectations and legal constraints. The study contributes to criminological theories of decriminalization and symbolic compliance by understanding, why and how, through their own communications, corporations systematically resist framing their misconduct in criminal terms and manage this resistance while navigating the institutional pressures of criminal proceedings.

Transnational corporate corruption is a sophisticated business strategy, executed in corporate offices and hidden behind offshore legal entities, layers of financial transfers, and multiple intermediaries. Multinational companies may bribe foreign public officials to win contracts, secure market access, or tilt regulations in their favor. These arrangements often span continents and decades, deliberately obscuring both the money trail and the people who profit from it (Lord, 2024). 

When transnational corporate corruption cases undergo judicial examination, they typically come to public attention through media outlets (Macfarlane Horn, 2024a; Tabbert, 2012), podcasts (Macfarlane Horn, 2024b), press releases from state authorities (Capus & Hohl Zürcher, 2024), and civil society organizations (Khambekova et al., 2021). Corporate self-disclosure adds a further dimension to this communicative field, raising the criminologically significant question of how offenders under scrutiny present their own wrongdoing. This choice to communicate publicly is not incidental: it is a strategic attempt to manage narratives, balance disclosure with denial, and protect corporate legitimacy at a moment of acute vulnerability. Yet how corporations frame and communicate their involvement in such corruption once criminal proceedings are underway is an understudied topic.

Corporate communication studies have highlighted the growing importance of corporate social responsibility (CSR) discourse (Ogrizek, 2002; Steyn, 2003), yet corruption remains under-acknowledged despite its systemic harms (Hess, 2009; Hess & Ford, 2008). Communication about corruption thus poses a particular dilemma: It threatens corporate reputation (Alexander, 1999; Gottschalk, 2013) and may jeopardize business relations, yet silence also entails reputational and legal risks. Existing work has shown that representations of corporate corruption vary across stakeholders (Khambekova et al., 2021; Macfarlane Horn, 2024a), and that external pressure is the primary driver of corporate disclosure (Blanc et al., 2017; Schranz et al., 2020). Prior studies of corporate communication (Blanc et al., 2019; de Andrés et al., 2023) have, however, approached disclosure from a business perspective rather than a criminological one, focusing on reputational repair and stakeholder management rather than on how corporations contest, contain, or selectively acknowledge their own criminal conduct.

This paper addresses that gap. Theoretically, it draws on two complementary frameworks. First, it builds on Tombs and Whyte’s (2015) analysis of corporate decriminalization, which is the active process through which corporations resist the criminal framing of their conduct across legislative, judicial, and cultural arenas. It argues that corporate communication constitutes a further, underexplored site of this process. Second, this study mobilizes Edelman’s (2016) concept of symbolic compliance to examine how corporations perform accountability at the institutional moments when it is unavoidable, while strategically retreating from it as pressure subsides. Together, these frameworks allow us to move beyond the question of whether corporations disclose their wrongdoing, toward the analytically richer question of how they do so.

Empirically, the study draws on press releases and annual reports issued by ten globally operating companies from a range of industries, all of which faced criminal proceedings for the bribery of foreign public officials resolved through a non-trial resolution (NTR) in Switzerland between 2011 and 2023. Through qualitative content analysis, we examine (1) the frequency and timing of corporate communication in relation to the criminal proceeding, (2) the framing strategies used to present the corruption event and the criminal procedure, and (3) the sequential dynamics of these strategies across the stages of proceedings.

Our findings show that corporate communication is overwhelmingly reactive, triggered by external pressure. We identify four distinct frames through which corporations present their wrongdoings — criminal, illegal, managerial, and righteous. They function as discursive instruments through which corporations selectively acknowledge, minimize, or deny their criminal conduct. Moreover, we document a sequential and dynamic logic in the deployment of these frames.

## Corporate corruption portrayals

Following Steyn (2003), corporate communication is best understood as an integral part of a broader corporate strategy. It results from deliberative processes involving communication professionals and senior management and is continuously adapted to changing conditions. It seeks to build and maintain relationships with stakeholders such as employees, customers, suppliers, investors, or civil society (Steyn, 2003, p. 180).

According to the few scientific studies on (anti)corruption communication, corporations maintain their relationships with stakeholders primarily by referring to anticorruption policies and compliance measures, rather than by disclosing actual corruption cases. Notably, Sari et al. (2021) examine how corporations disclose anticorruption policies. Their findings show that such disclosure varies significantly across firms and countries, and that institutional frameworks play a key role in shaping the extent of voluntary anti-corruption disclosure. Blanc et al. (2017), in a study including 105 firms in 23 different countries, demonstrate that the higher the freedom of the press in a country, the more extensive the anticorruption reporting will be, also linking disclosure to external pressure. de Andrés et al. (2024) show that banks involved in corruption tend to increase their level of disclosure once the event becomes public. By contrast, banks not implicated in scandals generally communicate more proactively about corruption, often highlighting their anticorruption policies and distancing themselves from tainted competitors. In this way, stressing competitors’ corruption while emphasizing their own compliance serves to “send a signal to stakeholders and investors concerning [their] reliability” (de Andrés et al., 2023, p. 121). This echoes the argument of Kasl Kollmannová (2013), who qualifies anticorruption discourse as a marketing tool that repairs the bad image of businesses, especially after the 2008 global financial crisis. This “masking” of bad behavior also manifests itself through the higher disclosure of ESG^1^ details (El Khoury et al., 2023; Kapardis et al., 2025).

Taken together, the literature shows that corporate communication about corruption is shaped primarily by external pressures—including institutional frameworks, press freedom, and the public exposure of corruption events—while anticorruption disclosure is used strategically to manage reputational risks and differentiate companies from corrupted counterparts. Whereas these works focus broadly on anticorruption policy^2^ communication and communication about corruption events, other studies investigate more specifically how companies respond when their own misconduct becomes public. Schranz et al. (2020) demonstrate that firms experiencing a “compliance-crisis”—that is, when their behavior regarding internal standards, industry guidelines, social values, or law is problematized in the media—tend to communicate less, or in a more passive manner. Such crises are mainly disclosed by external instances, such as authorities, the media, or non-governmental organizations. Where communication does occur, concrete crisis management measures (e.g., internal investigations, admissions of fault, apologies, employee dismissals, or compensation payments) are seldom addressed, raising the question of whether such measures are not undertaken or simply withheld from public discourse.

When corporations do engage in corruption crisis communication, however, their approach is highly strategic and audience specific. Blanc et al. (2019) show in a case study on Siemens that communication tends to intensify after the scandal becomes public, carefully tailored to restore legitimacy with different stakeholder groups. Notably, corruption is addressed largely through references to anticorruption guidelines rather than explicit discussion of the company’s wrongdoing. In brief, corporations use (anti)corruption communication mostly to signal integrity, restore legitimacy, and reassure stakeholders (de Andrés et al., 2024).

The existing literature on corporate communication related to corruption presents important limitations. First, it is mainly rooted in finance and business management studies and therefore focuses on the effectiveness of communication strategies and the restoration of corporate legitimacy rather than on the criminological dimensions of corporate corruption. This perspective tends to obscure the relationship between corporations and their offenses, favoring reputational repair over accountability. Moreover, research on corruption disclosure predominantly adopts quantitative approaches, measuring the frequency of disclosure rather than qualitatively analyzing its content. Yet the substance of communications—and the framing strategies through which they are presented—is crucial.

A more nuanced criminological perspective is therefore needed: one that analyzes not only whether corporations disclose corruption-related information, but also when and how they frame these disclosures, and which rhetorical strategies they deploy to manage their image when facing corruption allegations and criminal proceedings.

## Theoretical framework: corporate power and symbolic compliance

The existing literature reviewed above provides important insights into whether and how often corporations disclose corruption-related information, yet it remains largely silent on two interconnected questions: why corporations systematically resist framing their misconduct in criminal terms, and how they manage this resistance while navigating the institutional pressures of criminal proceedings. To address these questions, this study draws on two complementary bodies of criminological and socio-legal theory — Tombs and Whyte’s (2015) framework of corporate decriminalization and Edelman’s (2016) concept of symbolic compliance — and argues that corporate communication during criminal proceedings constitutes an underexplored site where both processes operate simultaneously.

### Corporate decriminalization as an active process

A foundational contribution of Tombs and Whyte’s (2015) is that the structure of the modern corporation renders criminal behavior not only possible but, under certain conditions, economically rational. Their central argument is that the legal and organizational form of the corporation — characterized by limited liability, the multiplication of subsidiary entities, and the structural separation between ownership and management — produces what they term “structured irresponsibility”: a systematic diffusion of accountability that makes it structurally difficult to attribute criminal responsibility to any identifiable individual or entity. Crucially, the authors insist that the decriminalization of corporate conduct is not a passive absence of enforcement but an active process, sustained at multiple levels simultaneously — legislative, judicial, mediatic, and cultural.

Central to this process is the synoptic dimension of corporate power: corporations actively manage their own public visibility, projecting carefully curated self-images that naturalize their presence and legitimacy in social life. The public relations and corporate communication industries are, in their view, central instruments through which corporations assert their socially necessary and beneficial role. From this perspective, corporate communication about criminal proceedings is not merely a reputational strategy; it is a form of cultural power through which the boundaries of criminal accountability are actively contested.

Tombs and Whyte’s framework, however, primarily document how legislatures, regulatory agencies, and media contribute to corporate decriminalization, they do not examine systematically how corporations themselves participate in this process through their own discursive practices. This gap is significant: if decriminalization is a multi-level and multi-actor process, the offending corporation’s own voice is a key site of analysis that has received insufficient criminological attention.

### Symbolic compliance and the performative management of accountability

The second theoretical resource mobilized in this study is Edelman’s (2016) concept of symbolic compliance. Edelman demonstrates that organizations respond to legal mandates not primarily by altering their substantive practices, but by constructing compliance structures — codes of conduct, ethics committees, internal investigation procedures, diversity offices — that signal adherence to legal norms without necessarily producing their intended effects. Over time, these structures come to be accepted by courts and regulators themselves as evidence of good faith. The result is a form of accountability that is, in Edelman’s terms, more symbolic than substantive.

Edelman’s framework illuminates an important dimension of corporate behavior during criminal proceedings: the deployment of compliance rhetoric — references to zero-tolerance policies, enhanced internal controls, cooperation with authorities, and reformed governance structures — as a means of satisfying institutional expectations while limiting the symbolic and reputational costs of criminal acknowledgment. Yet Edelman’s analysis focuses primarily on stable organizational structures rather than on sequential discursive practices, and it does not specifically address the context of criminal proceedings, where institutional pressure is at its most acute. These are precisely the conditions under which the temporal and strategic dimensions of symbolic compliance become most visible.

### Toward a criminology of offender-driven communication management

Taken together, Tombs and Whyte’s decriminalization framework and Edelman’s concept of symbolic compliance offer complementary but distinct analytical resources. What neither framework provides, however, is an empirically grounded, temporally situated account of how these dynamics unfold in practice across the stages of a criminal proceeding, as seen through the corporations’ own communications. This is the specific contribution of the present study: by analyzing how corporations frame their conduct before, during, and after criminal proceedings, and by identifying the sequential logic of these framing strategies, we document the discursive mechanisms through which corporate decriminalization and symbolic compliance operate — a domain that has yet to be systematically explored.

## Research strategy

To address this gap, the study examines how companies communicate about bribery of foreign public officials and the corresponding criminal proceedings.^3^ Rather than examining the corruption cases themselves, we focus on the communicative strategies companies employ to frame these incidents — what Hopwood (2009) calls the “corporate veil”, understood as the rhetorical layer a company places over internal misconduct. Since framing operates precisely through selective emphasis and omission, it offers an established approach to making this veil analytically visible. Following Entman (1993), framing is understood as the method of choosing and highlighting specific real-world elements in written communication to reinforce desired narratives about problems, causation, ethics, and appropriate responses — thereby revealing which aspects of a case a company foregrounds, downplays, or omits entirely. Understanding how an event is framed is important because the perception of an event determines how it is problematized and, consequently, how the problem must then be solved (Best, 2017).

The concept of framing has already been helpful in criminological research to understand how media (Macfarlane Horn, 2024a) or civil society organizations (Khambekova et al., 2021) portray corruption. Both studies show that the understanding and problematization of corruption can vary. These variations influence the potential action taken against corruption. For example, Khambekova et al. (2021) found that civil society organizations with a better understanding of corruption deploy more efficient actions to fight against it. However, so far, the focus is on framing used by third-party actors. In the present study, we investigate the main player: the offending company.

This article specifically addresses the following research questions: *(1) How frequently do corporations disclose their own corruption cases, and at what stage of the criminal proceedings does this occur? (2) What framing strategies do corporations employ to present the corruption event and the criminal procedure? (3) In what sequence do these framing strategies appear over the course of the proceedings?*

### Case selection and limitations

This article adopts a qualitative, exploratory research design to examine how companies time and frame their involvement in corruption and the ensuing criminal proceedings.

The study is based on the corporate communications of ten globally operating companies, all of which were implicated in a corruption case resolved through a non-trial resolution (NTR) in Switzerland between 2011 and 2023. Switzerland represents a pertinent case study for several reasons. Unlike most existing studies, which focus on common law jurisdictions, Switzerland offers a civil law perspective, broadening the comparative scope of research on corporate responses to criminal proceedings. As a jurisdiction hosting major corporate headquarters and significant financial activities, it also provides a rich terrain for examining such responses. The Swiss system of non-trial resolutions (NTR) shares the same core features as their common law equivalents, particularly the opacity of proceedings, lending our findings broader comparative relevance. Crucially, the companies involved are largely transnational corporations. Switzerland thus functions less as a national case study than as a lens through which the communicative dynamics of transnational corporate crime enforcement can be examined in a focused and bounded way.

This sample constitutes a complete census of all companies against which a criminal ruling for the bribery of foreign public officials was issued in Switzerland during that period; the temporal boundaries are therefore determined by the availability of cases rather than by a principled selection decision. The corporations involved are distributed across various industries, including construction, engineering, and commodity trading, and are headquartered in several countries, predominantly in Europe. Table 1 summarizes the cases relevant to this study.

**Table 1:** Overview of cases

Corporation^a^	Industry	Headed in	Year of NTR’s conclusion in Switzerland^b^	Financial consequences in Swiss Francs^c^
ABB	Engineering	Switzerland (CH)	2022	4.1 million
Alstom	Engineering	CH (convicted company); France (group)	2011	39.0 million
Andrade Gutierrez	Construction	Brazil	2019	18.7 million
DEME	Construction	Cyprus (convicted company); Belgium (group)	2017	37.8 million
Gunvor	Commodity trade	CH	2019	93.7 million
Koenig & Bauer	Engineering	CH (convicted company); Germany (group)	2017	35.0 million
Ameropa	Agricultural trade	CH	2016	0.9 million
Odebrecht	Construction	Brazil	2016	> 200 million
SBM Offshore	Oil and gas industry	CH (convicted companies); the Netherlands (group)	2021	7.0 million
Sicpa	Chemical industry	CH	2023	81.0 million

^a^The name refers to the corporation in whose name the annual reports and press releases are issued. In most cases, these are corporate groups. However, the companies convicted in Swiss criminal proceedings are often subsidiaries. Since these subsidiaries typically do not have their own communication channels, and this study focuses on communication rather than legal responsibility, the names of the parent companies are used

^b^The Swiss NTR was established in coordination with other prosecution authorities in the following cases: ABB (additional countries involved: South Africa, the US), Odebrecht (the US, Brazil), Andrade Gutierrez (Brazil), SBM Offshore (the Netherlands, the US, Brazil)

^c^Financial consequences refer to the amount of money the firm will have to pay to Switzerland and includes the criminal fine, confiscated assets, substituting assets if the assets subject to forfeiture are no longer available, and costs of proceedings

NTRs have been adopted in at least 27 OECD member countries, among them the United States, the United Kingdom, France, the Netherlands, and Switzerland (OECD, 2019, pp. 11–12). While NTRs vary procedurally across jurisdictions — notably in whether they result in a formal conviction — they share the defining feature of allowing criminal cases to be concluded without a full court proceeding, typically through a negotiated agreement between the company and the prosecuting authority (OECD, 2019, p. 3). The NTR instrument applied in Switzerland is the Summary Penalty Order (SPO),^4^ a conviction-based mechanism administered by the Office of the Attorney General of Switzerland (OAG)^5^ (OECD, 2019, pp. 27, 55). Under Swiss law, corporations are convicted for failing to take all reasonable organizational measures to prevent the bribery of foreign public officials (Article 102, Paragraph 2 of the Swiss Criminal Code).

In transnational corruption cases, NTRs are frequently embedded in broader global resolutions in which proceedings across multiple jurisdictions are concluded through a coordinated effort involving different prosecuting authorities. This applies to four of the ten companies in this study — ABB, Andrade Gutierrez, Odebrecht, and SBM Offshore — whose corporate communications address not only conduct prosecuted in Switzerland but also related proceedings in other jurisdictions, including the United States, Brazil, and South Africa. The inclusion of these cases does not compromise analytical coherence; on the contrary, it ensures that the sample reflects the full spectrum of transnational corporate corruption cases, from those resolved within a single jurisdiction to those embedded in complex multi-jurisdictional resolutions.

Focusing on NTR cases is particularly pertinent for two reasons. First, NTRs have become the predominant instrument for resolving transnational corporate corruption cases. Second, NTRs, by design, preclude a full public trial — making corporate communications an important source through which the public narrative of such cases might be shaped.

The study’s sample has two notable limitations. First, with ten cases, the sample is small, and findings should be interpreted with caution regarding their generalizability. The study is best understood as an exploratory contribution that lays the groundwork for larger-scale investigations encompassing a broader range of companies and jurisdictions. Second, the sample is limited to cases resolved through NTRs. Because NTRs are negotiated confidentially rather than litigated in public hearings, what the public learns about a company’s criminal conduct depends largely on the disclosure of both the company and the prosecuting authority.

### Materials

The material for this study consists of press releases and annual reports, as these corporate communication instruments are considered highly relevant for a broad public audience, employees, suppliers, and investors (Blanc et al., 2017; de Andrés et al., 2023).^6^ The press releases and annual reports included in the study are publicly available on the corporations’ websites and were downloaded as PDF files.^7^ While annual reports are extensive, ranging from 94 to 304 pages, press releases are at most two pages long.

To examine corporate communication and its potential changes over the course of criminal proceedings — as addressed by research questions one and three — the publication period considered corresponds to the duration of the Swiss criminal proceedings plus and minus one year. The preceding year is included because companies are typically already aware of a corruption event before formal criminal proceedings begin and may conduct internal investigations, take personnel decisions, or engage in other actions that could result in public communication. The subsequent year is included to account for the fact that annual reports are produced retrospectively and may reference proceedings that have recently concluded. Where a corporation faced prosecution in multiple countries, the duration of the Swiss proceedings serves as the reference period, ensuring a consistent temporal framework across all cases.^8^

Across all publication periods, 2,745 documents were pre-selected, comprising 2,729 press releases and 16 annual reports. Among these, documents containing communications related to the corruption event addressed by the Swiss criminal proceeding were selected for analysis, based on an extensive keyword search. This search included general keywords relating to corruption and criminal proceedings — such as bribe* (B/bestech* in German; *pot-de-vin*, corromp* in French) or *Office of the Attorney General* (*Bundesanwaltschaft*; Ministère public*) — as well as case-specific keywords, such as names of countries involved in the corruption (e.g., Latvia, Tunisia, and Malaysia in the case of Alstom), names of projects (e.g., *Kusile* in the case of ABB), or terms typically used by the corporation to refer to the NTR proceedings (e.g., *leniency agreement* in the case of Odebrecht).

Since annual reports, in general, contain multiple mentions of corruption across different sections, each mention was counted separately as a single citation. The final sample consists of 55 citations (*n*_c_) distributed across 28 documents (*n*_*d*_): 40 citations (*n*_*c*_) identified in annual reports and 15 citations, in press releases. Table 2 provides an overview of press releases, annual reports, and citations per case.

**Table 2:** Overview of press releases and annual reports published during the Swiss criminal proceedings related to corruption and organizational deficiencies against the corporations (Plus/Minus One Year) as well as of citations mentioning them

Corporation	Period of analysis	Press releases	Press releases	Annual reports	Annual reports	Citations found in PR and AR	Type of company^d^
		Total *N*	Selected *n*_*d*_	Total *N*	Selected *n*_*d*_	*n*_*c*_	
ABB	10/2021–12/2023	174	5	2	2	12	Public company
Alstom	07/2008–11/2012	1,957	2	4	4	11	Public company
Andrade Gutierrez	11/2017–11/2020	Not available		Not available		n/a	Private company
DEME	01/2011–05/2018	Not available		Not fully available		n/a	Public company
Gunvor	05/2016–10/2020	11 (the period is not fully covered)	0	Not available		n/a	Private company
Koenig & Bauer	12/2014–03/2018	4 (only regulated information accessible, not press releases)	1	4	2	8	Public company
Ameropa	01/2011–05/2017	Not fully available		Not available		n/a	Private company
Odebrecht	07/2014–12/2017	365	3	3	3	12	Private company
SBM Offshore	09/2019–11/2022	97	2	3	2	10	Public company
Sicpa	01/2014–04/2024	121	2	Not available		2	Private company
Total		2,729	15	16	13	55	

*N* = total number of press releases or annual reports published in the relevant period of analysis; *n*_*d*_ = number of documents relevant for this study—that is, press releases or annual reports containing information on the crime prosecuted in Switzerland or its related proceeding; *n*_*c*_ = number of single citations found in the press releases and/or annual reports

^d^The classification of companies is based on the timing of the conclusion of criminal proceedings in Switzerland. A public company offers its shares on stock markets to the general public, whereas a private company is owned by a limited group and its shares are not publicly traded; for further details, see, e.g., https://www.investopedia.com/terms/p/publiccompany.asp and https://www.investopedia.com/terms/p/privatecompany.asp (last visit: 18th September 2025)

### Data analysis

The QDA software Atlas.ti was used. A qualitative content analysis inspired by the content structuring approach (Kuckartz, 2018) helped to identify relevant themes and sub-themes through a combined deductive-inductive approach and to build a coding scheme. Concretely, the deductive strand allowed us to search for topics in the material based on literature on corruption, such as crime, perpetrator/offender, victim, or NTR proceedings. Conversely, the inductive strand allowed a material-based expansion of this a priori defined range of topics. It is through reading and rereading the material that new topics emerge (Thomas, 2006). It particularly allowed us to identify the following four frames: the criminal, the illegal, the managerial, and the righteous frame. Before the four frames are described in detail, the following section first addresses general differences in corporate communication across cases, followed by findings pertaining to the first research question on the frequency and timing of corporate disclosures.

## Results

### General pattern in corporate communication

As reflected in the varying number of published documents prior to selection (see Table 2), the corporations examined in this study followed markedly different communication strategies. Some corporations — Alstom, Koenig & Bauer, SBM Offshore, ABB, and Odebrecht — communicated regularly through both channels, press releases and annual reports. SICPA limited its communication to press releases, as its annual reports were not publicly accessible. Three other companies — Andrade Gutierrez, Ameropa, and DEME — produced little or no accessible communication during the period of interest. Finally, Gunvor appears to have communicated primarily through press releases from 2009 onwards; however, press releases from three relevant years (2017–2019) are absent from its website. Due to insufficient data, Andrade Gutierrez, Ameropa, DEME, and Gunvor could not be coded and analyzed. However, the absence of publicly accessible documentation is in itself an analytically relevant finding. 

The corporations most active in communication are publicly listed companies — Alstom, Koenig & Bauer, SBM Offshore, and ABB — as well as Odebrecht, a family-owned firm that was not only among the largest employers in Brazil but also a central actor in the corruption scandal surrounding the semi-state-owned company Petrobras, which attracted considerable public attention both in Brazil and internationally.^9^ The less communicative corporations are predominantly privately owned. This pattern of communication is mirrored by the communication strategies of public authorities: companies with no or minimal accessible communication such as Andrade Gutierrez, Ameropa, or DEME are precisely those whose NTR conclusions were either not communicated by the Swiss criminal justice authority or disclosed only in anonymized form (Capus & Hohl Zürcher, 2024, pp. 11, 17).

These findings align with prior research indicating that corporate communication is largely driven by external pressures (e.g., media, public, or NGOs), rather than by voluntary disclosure (Blanc et al., 2017; Sari et al., 2021). Publicly listed corporations face institutionalized communication obligations — including mandatory disclosure requirements under stock exchange regulations^10^ — that compel transparency regardless of reputational preferences. Privately owned companies, by contrast, operate under no such obligation and appear largely unresponsive to public scrutiny, particularly in the absence of disclosure by other parties such as criminal justice authorities. Taken together, this initial analysis supports the argument that corporations communicate when they must, rather than by choice.

### Frequency and timing of corporate disclosures

Addressing the first research question — how frequently corporations disclose their own corruption case and at what stage of the criminal proceeding this occurs — this following section examines the number of documents published and their distribution across the stages of the Swiss criminal proceedings.

**Fig. 1:**
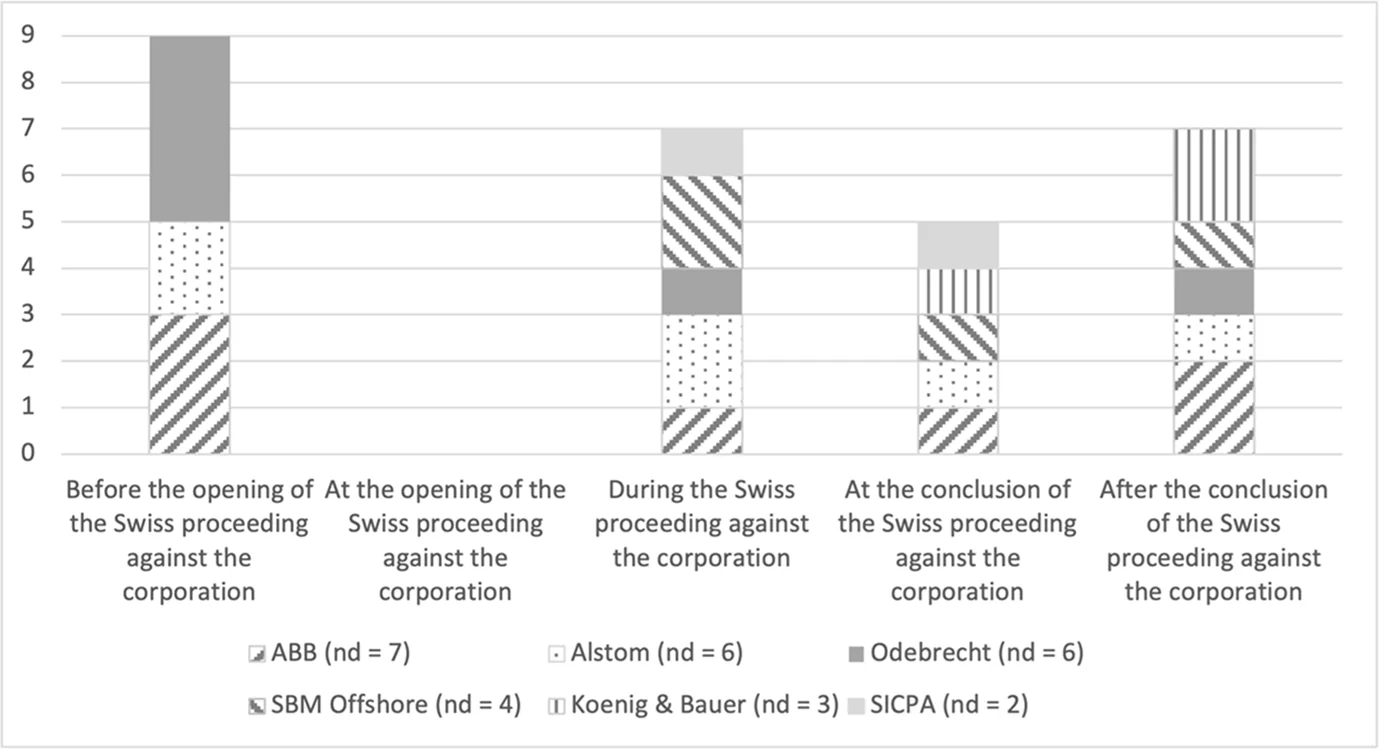
Number of documents mentioning the corruption case, by corporation and stage of criminal proceedings (press releases and annual reports; n_d_ = 28)

The 28 documents identified across all cases show that most corporations disclosed their corruption cases at multiple points in time — before, during, and after the Swiss criminal proceedings, see Fig. 1. For each corporation, Fig. 1 illustrates the number of documents mentioning the corruption case and the stage of the criminal proceedings at which they were issued. Only one corporation communicated exclusively at the conclusion of the proceedings and thereafter (Koenig & Bauer). This variation points to the role of external factors in driving disclosure decisions, which can be grouped into three distinct triggers.

The first and most prominent triggers are the procedural decisions. At the conclusion of the Swiss proceedings, corporate communication most often takes the form of press releases announcing the resolution of the case (*n*_*d*_ = 5). Notably, the headlines of these press releases do not always make the criminal nature of the proceedings explicit, as illustrated by the following examples^11^:

SICPA Press Release About OAG (SICPA, press release, 27.4.2023)

ABB concludes settlements for Kusile project (ABB, press release, 2.12.2022)

The Swiss Office of Attorney General confirms the absence of any bribery system at Alstom. It fines the company for “corporate negligence” in three isolated cases (Alstom, press release, 22.11.2011)

Conclusion of legacy issue in Switzerland (SBM Offshore, press release, 23.11.2021)

Procedural decisions in foreign jurisdictions involving the same corruption case also function as triggers for communication prior to and during the Swiss proceedings. ABB, for instance, reported on the conclusion of criminal proceedings with South African authorities, detailing associated financial measures while simultaneously referring to the Swiss proceedings in vague terms („various authorities in other countries “; Annual Report 2021, p. 213; Press Releases, 3.2.2022, 30.9.2022, 20.10.2022). Similarly, SBM Offshore used a deadline related to NTR proceedings in the United States as an occasion to retrospectively disclose the initiation of the Swiss proceedings (Press Release, 30.11.2020; Annual Report 2020, pp. 150, 196). The significance of foreign proceedings as a communication trigger is underscored by the Koenig & Bauer case — a case prosecuted exclusively by Swiss authorities — in which corporate communication occurred only upon the conclusion of the Swiss proceedings (see Fig. 1).

The second trigger is regulation. For publicly listed companies, stock exchange regulations require the disclosure of significant legal cases and their financial impact, resulting in information on provisions, reduced earnings, and governance adjustments being published through annual reports or press releases accompanying quarterly or annual results also during or after the conclusion of the criminal proceedings.

The third trigger is public and media pressure. Two cases illustrate this dynamic. In the Odebrecht case, publicly disclosed allegations prompted the company to issue three press statements shortly before the Swiss proceedings commenced (May 2015) and to include a “Message from the Chairman of the Board” in its annual report written in the same period (Annual Report 2014/5, p. 10ff), in which the corporation strongly denied the corruption allegations. The tone, however, shifted markedly in the subsequent annual report (2015/6, pp. 12f., 14f., 31), published after the arrest of five executives, first-instance convictions in Brazil, and significant changes in senior management — developments that rendered the company's involvement in the scandal undeniable. Alstom’s communication prior to and during the Swiss proceedings similarly reflects media pressure: the company issued, for example, a press release explicitly responding to allegations published by the *Wall Street Journal.*^12^ This press release, titled “Alstom to Take Legal Action to Protect Its Reputation” (15.9.2008), categorically denied all allegations and announced its intention to pursue legal action against the outlet.

Taken together, these findings indicate that corporate disclosure is largely reactive rather than voluntary. Corporations communicate in response to procedural decisions by judicial authorities — in Switzerland or abroad — market regulation, and public or media attention. In the absence of such pressures, the default tendency is silence.

### Framing of corporate corruption

**Fig. 2:**
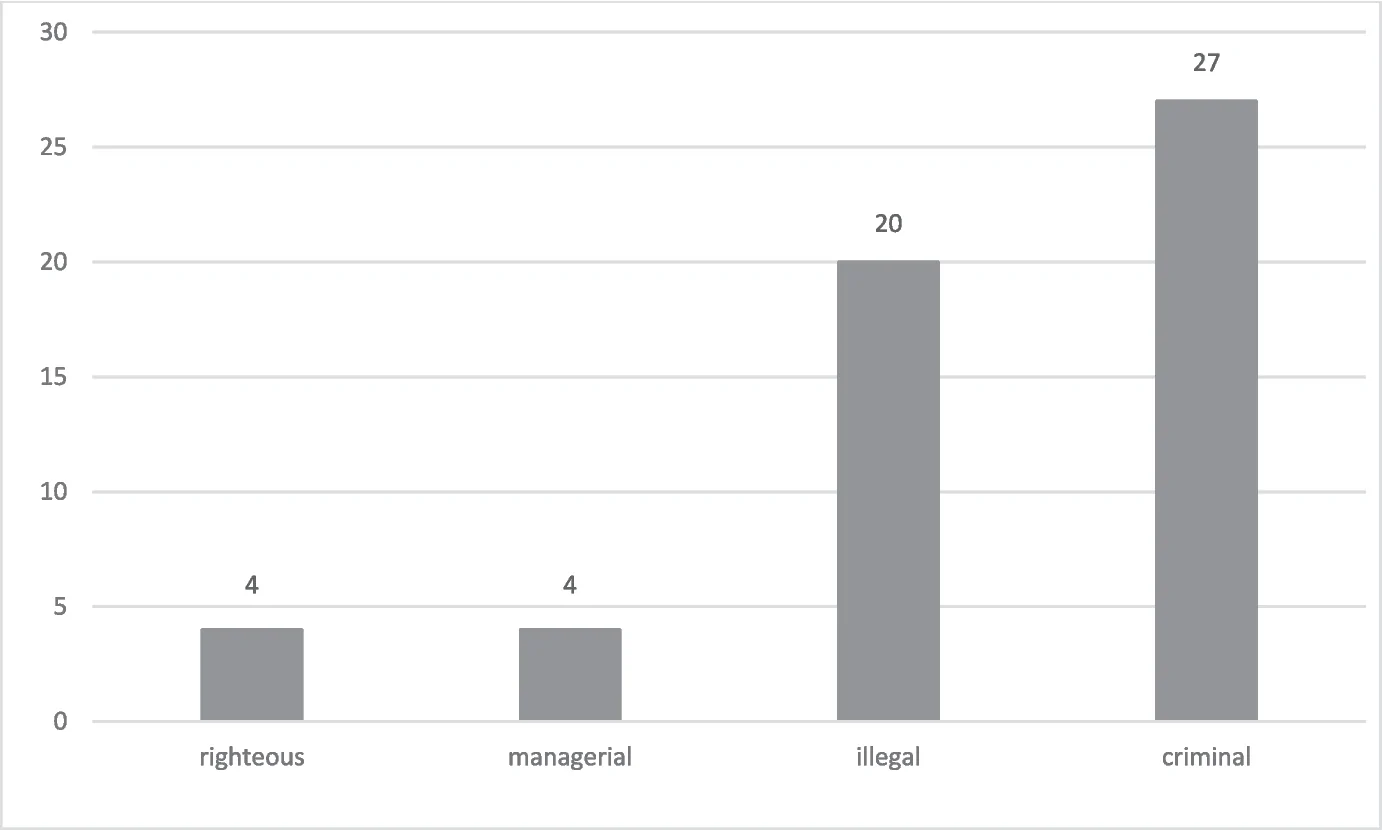
Distribution of communications by frame (n_c_ = 55)

Despite a common tendency toward vagueness when communicating about wrongdoing, the corporations studied frame their corruption cases in markedly different ways, foregrounding certain elements while ignoring or nuancing others to present their involvement in corruption under various lights (Best, 2017; Entman, 1993). Addressing the second research question — what framing strategies corporations employ to present the corruption event and the criminal proceedings — four distinct frames are identified: the *criminal frame*, in which the chosen terminology portrays the corruption event as criminal behaviour, referencing concepts such as bribery, corruption, or criminal justice authorities; the *illegal frame*, in which the conduct is cast as a violation of laws (but not explicitly criminal law) or regulatory requirements, without invoking criminal justice concepts; the *managerial frame*, in which the language situates the conduct within the realm of internal governance or compliance, stripped of any legal or criminal connotation; and the *righteous frame*, in which corporations present themselves as ethical and compliant actors, distancing themselves from any wrongdoing. The criminal and illegal frames are substantially more prevalent than the other two (see Fig. 2).

#### Criminal frame

The criminal frame encompasses two distinct sub-frames: the *criminal-repressive* frame, which portrays the proceedings as conventional criminal proceedings punishing a criminal act, and the *criminal-consensual* frame, which acknowledges the criminal nature of the event but emphasizes the consensual manner in which it is resolved, with the company cast in a proactive role.

The criminal-repressive frame is the dominant communicative style, accounting for 17 of the 27 citations identified as criminal frames. In these citations, corporations portray corruption and the associated proceedings primarily as what they are in legal terms: criminal conduct subject to prosecution and sanctions by judicial authorities. A typical example is the following press release, in which a company announced the conclusion of the Swiss criminal proceedings (SBM Offshore, Press Release, 23 November 2021, emphasis by the authors):

In November 2020, the Company communicated that three of the Company’s subsidiaries in Switzerland received a notification from the **Bundesanwaltschaft (Public Prosecutor Office)** in Bern. It concerned a **suspicion** that from 2005 till 2012 these subsidiaries failed to take all reasonable and necessary organizational measures to prevent the commission of acts of active **bribery** of foreign public officials during said period.

On this matter, the **Swiss public prosecutor** has now issued an **investigation termination order** and a **criminal penalty order** against the three Swiss subsidiaries, requiring them to pay a total amount of 7.0 million CHF (c. USD 7.6 million).

[Name], Chief Governance and Compliance Officer said: “The fact pattern and compliance shortcomings prior to 2012 that led to the **Swiss penalty** are also covered by the legacy resolutions the Company concluded in the Netherlands (2014), the United States (2017), and Brazil (2018). The termination of the investigation and penalty now also close this old issue in Switzerland on a full and final basis. Today and for many years now, the Company has been implementing substantial measures to ensure that it operates with integrity and fully in line with laws, regulations and with its compliance standards. These measures were also recognized by the **Swiss Public Prosecutor Office**.”

The combination of terms such as “suspicion”, “bribery”, “criminal penalty order”, and “public prosecutor office” clearly signals that the company is reporting on criminal conduct and the conclusion of criminal proceedings. Of particular analytical interest is the dual-language reference to the Swiss authority: by providing both the German designation (*Bundesanwaltschaft*) and its English translation (*Public Prosecutor’s Office*), the company signals to an international lay audience that it is indeed a criminal justice authority.^13^ At the same time, although the proceeding constitute an NTR, the company presents it as a conventional criminal procedure in which the judicial authority unilaterally investigates and adjudicates the matter.

The remaining 10 citations correspond to the criminal-consensual frame. Here too, the corruption event is presented in criminal law terms, but the companies explicitly foreground the consensual nature of the proceedings. A typical example is the following press release by ABB (2 December 2022):

**ABB concludes settlements for Kusile project**

*Settlement of $327 million already announced and accounted for in the third quarter 2022 financial statements.*

ABB has reached a full and final settlement with the National Director of Public Prosecution in South Africa, the U.S. Department of Justice, the U.S. Securities and Exchange Commission, and the Office of the Attorney General of Switzerland related to the legacy Kusile project in South Africa, awarded in 2015.

ABB hopes to reach a final resolution with German authorities in the near term.

[…]

ABB CEO Björn Rosengren: “We take the Kusile matter very seriously. Since it was reported, ABB has cooperated fully with all authorities and spent considerable time and effort - including launching a new code of conduct, educating employees and implementing an enhanced control system - to prevent something similar from happening again. We have a clear zero tolerance approach to non-ethical behavior within our company.

Both the headline “ABB concludes settlements for Kusile project” and the statement “ABB has reached a full and final settlement” cast the company in an active, agentic role, framing the criminal proceedings not as a unilateral decision imposed by the authorities but as a mutually negotiated outcome. A similar strategy is evident in Koenig & Bauer’s Supervisory Board statement, published in the introduction of its 2017 annual report (p. 6):

In a conference call on 20 February 2017, we approved the proposed settlement with the Swiss Office of the Attorney General to resolve the self-disclosure proceedings of KBA-NotaSys SA regarding deficiencies in the prevention of corruption.

Although the explicit mention of the Swiss Office of the Attorney General clearly identifies the proceedings as criminal in nature, both the term “self-disclosure proceedings” — a formulation coined by the company itself — and “settlement” strategically foreground the company’s active and voluntary role: the former implies that the company proactively initiated the process, while the latter frames the outcome as a mutually negotiated agreement rather than a unilaterally imposed decision.

In sum, both criminal sub-frames portray the corporation’s conduct as criminal behavior; what differs is the posture of the company. In the criminal-repressive frame, the company appears as a passive actor subject to the authority of the criminal justice system — a posture that conveys acceptance of responsibility without foregrounding the company’s agency. In the criminal-consensual frame, the company presents itself as a proactive entity working toward justice in collaboration with the authorities, conveying the implicit message that the criminal conduct belongs to the past and that the corporation has actively worked to bring it to an end.

#### Illegal frame

Whereas the criminal frame employs the terminology of criminal law, the illegal frame is defined precisely by its absence. A total of 20 citations reflect this framing strategy, in which corporations acknowledge legal violations without ever characterizing them as criminal conduct — neither regarding the offense itself, the proceedings, nor the authorities involved.

The offenses for which the companies were sanctioned—specifically, organizational failures to prevent corruption—are not characterized as criminal conduct. Instead, corporations employ euphemistic or deliberately ambiguous formulations, such as “illicit payment claims” (e.g., Alstom, Annual Reports 2010, 2011, 2012), “potential suspect payments” (e.g., ABB, Annual Report 2022, p. 213), or “illegal and illegitimate system” and “practices that are harmful to one’s self and society as a whole,” as illustrated in the following example:

Although we had no dominant responsibility for the **facts established in Operation Car Wash**, we were involved in an **illegal and illegitimate system of political-electoral financing**. The heavy construction industry, due to the complexity of the projects and the amount of resources involved and the proximity between public and private agents for their execution, is highly targeted by the public sector. This sometimes results in acquiescence with **practices that are harmful to one’s self and society as a whole**. (Odebrecht, Annual Report 2016, p. 12, Message from the Chairman of the Board; emphasis by the authors)‬‬‬‬‬‬‬‬‬‬‬‬‬‬‬‬‬‬‬‬‬

The same avoidance strategy extends to the proceedings and the authorities involved. Criminal proceedings are referred to in non-criminal terms — as “proceedings,” “public examination,” or “investigation” — while criminal justice authorities, such as the Swiss Office of the Attorney General and other criminal justice authorities, are reduced to generic “authorities” or “public authorities,” as in the following three examples:

South African, Swiss and American authorities (ABB, Annual Report 2022, pp. 9, 21, 112)

Swiss and French authorities (Alstom, Annual Report 2009, p. 115)

[…] public authorities in Brazil, the United States and Switzerland (Odebrecht, Annual Report 2017, p. 15)

The strategic function of this illegal frame lies in its capacity to de-dramatize the case: by systematically dissociating the conduct, the proceedings, and the authorities from the terminology of criminal law, corporations reduce the perceived severity of the wrongdoing. In this way, for audiences such as investors and other stakeholders, the illegal frame conveys an image of regulatory non-compliance rather than criminal misconduct, thereby minimizing reputational exposure while still formally acknowledging that misconduct occurred.

The criminal and illegal frames together account for 47 of the 55 citations in the sample (see Fig. 2). The two remaining frames — the managerial and the righteous — are considerably less frequent, each appearing in four citations, suggesting that most corporations stop short of fully neutralizing or denying their involvement in corruption.

#### Managerial frame

Where the criminal and illegal frames still acknowledge — explicitly or implicitly — that a legal violation has occurred, the managerial frame removes this dimension entirely. In four citations, corporations present the corruption event not as criminal or even illegal conduct, but as a managerial shortcoming: an operational or strategic mistake that has since been addressed.

This framing is most evident in communications related to financial performance, which are typically brief and contain no explicit reference to criminal proceedings or legal violations. Expressions such as “remaining matters for the legacy Kusile^14^ project” (ABB, Press Release, 20 October 2022; similarly, Annual Report 2022, p. 110), “mistakes made in the past,” or “our Group’s involvement in such episodes” (Odebrecht, Annual Report 2016, pp. 12–13) are used in ways that leave it to the informed reader to identify these as references to actual corruption cases. The term “legacy” — chosen not only by ABB but also by other corporations — is particularly telling: ordinarily associated with something neutral or even positive, it strips the underlying conduct of any criminal or moral connotation.

This stands in marked contrast to the typically detailed corporate governance sections in the same annual reports, where anticorruption efforts are explicitly labelled as efforts to prevent *corruption* and *bribery*. In the managerial frame, by contrast, the conduct is recast as part of broader processes of financial adjustment and strategic restructuring. The implicit message is that the issue lies in outdated practices or strategic missteps rather than in criminal or illegal reprehensible conduct — a technical problem that has been identified, resolved, and is unlikely to recur. The effect is to preserve reputational capital by presenting the corporation as a competent, self-correcting organisation rather than a criminal actor.

#### Righteous frame

While the previous three frames all acknowledge — to varying degrees — that something went wrong, the righteous frame represents a qualitatively different communicative strategy: rather than characterizing the conduct as criminal, illegal, or merely a managerial shortcoming, corporations employing this frame explicitly deny the allegations and present their conduct as ethical and legitimate.

In four communications published prior to or during the Swiss criminal proceedings, this framing is most prominently evident in the case of Odebrecht. In May 2015, during the ongoing Brazilian criminal investigation and shortly before the arrest of five executives, the company issued three public statements in which Odebrecht or its senior representatives categorically denied the corruption allegations and emphasized the legitimacy of the company’s conduct. The chairman of the Board of Directors, for instance, stated (Odebrecht, Annual Report 2015, p. 11):

We believe that our relationship with the government is completely legitimate-not only in Brazil but in all the countries where we operate.

Equally revealing is the statement of the company’s CEO — later convicted — who not only defended Odebrecht’s financing of the Brazilian president's travels but framed the entire matter as a question of societal trust (Odebrecht, Press Release, 1 May 2015):

Moving towards the construction of a society of trust based on respect between businesses, between businesses and the government, and between the government and the public would be very good for all of us.

This righteous posture proves to be unsustainable once NTR proceedings are formally concluded and made public by the criminal justice authorities. At that point, the frame is systematically abandoned — a pattern that underscores its essentially defensive and temporary character: it functions as a holding strategy in the face of unproven allegations, but collapses under the weight of a formal criminal conviction.

### Dynamic adoption of frames

Addressing the third research question — in what sequences framing strategies appear over the course of the proceedings — the analysis reveals that corporations employ various communication strategies, often using multiple frames simultaneously. Nevertheless, a consistent pattern emerges across cases (see Table 3): corporations tend to downplay the corruption event before and during the proceedings, only to adopt a criminal frame at the moment of conclusion, before gradually reintroducing softening frames thereafter.

**Table 3:** Frames employed according to Swiss criminal procedural stage for each corporation

	Before	Opening	During	Conclusion	After
ABB	Criminal, illegal, managerial		Managerial	Criminal	Criminal, illegal, managerial
Alstom	Illegal, righteous		Illegal	Criminal	Criminal, illegal
Koenig & Bauer				Criminal	Criminal, illegal
Odebrecht	Illegal, righteous		Criminal		Criminal, illegal
SBM Offshore			Criminal	Criminal	Criminal, illegal
SICPA			Criminal	Criminal	

This pattern is most likely driven by the public statements issued by criminal justice authorities upon the conclusion of NTR proceedings, which compel corporations to align their messaging with the official narrative and render outright denial or minimization untenable. In subsequent communications, corporations continue to employ the criminal frame but strategically combine it with illegal or managerial frames, maintaining a superficial alignment with official narratives while at the same time moderating the perceived severity of their conduct.

The case of Alstom illustrates this trajectory most clearly. Before the proceedings, the company communicated using a mix of illegal and righteous frames, simultaneously acknowledging some form of wrongdoing in non-criminal terms while denying that anything fundamentally illegitimate had occurred. During the proceedings, it shifted to the illegal frame exclusively, acknowledging wrongdoing without invoking criminal justice terminology. At the conclusion of the proceedings, it aligned with the criminal frame — consistent with the public announcement by the Swiss authorities — before reintroducing the illegal frame alongside it in subsequent communications.

The other corporations follow comparable trajectories, with one significant variation: for corporations that had already concluded NTR proceedings with foreign criminal justice authorities prior to the Swiss proceedings, the criminal frame appears earlier — not only at the conclusion of the Swiss proceedings. ABB, Odebrecht, and SBM Offshore fall into this category. Odebrecht, for instance, acknowledged the criminal nature of its conduct relatively early in the Swiss proceedings, despite having initially adopted a strong righteous posture (see Frequency and Timing of Corporate Disclosures). ABB began with a criminal frame due to the conclusion of the South African criminal proceeding nuanced by other frames, shifted toward the managerial frame, and then returned to the criminal frame at the conclusion of the Swiss criminal proceedings (see Criminal Frame). From the outset of the Swiss proceedings, SBM Offshore consistently employed a criminal frame—reflecting its previously concluded foreign NTRs—while softening it after the Swiss NTR by combining it with an illegal frame (see Frequency and Timing of Corporate Disclosures**)**. Alstom and Koenig & Bauer, by contrast, were prosecuted exclusively by Swiss authorities, with no prior foreign resolutions to compel earlier disclosure — consistent with both corporations adopting the criminal frame only at the conclusion of the Swiss proceedings.

While Alstom provides the clearest illustration of the overarching pattern, the case-specific variations observed across the other corporations further underscore a key finding: corporate framing strategies are largely reactive rather than pre-determined. Rather than following a fixed communication script, corporations adapt their framing at each stage of the proceedings in response to the constraints of their environment — including authority statements, media attention, and the expectations of investors and other stakeholders. The conclusion of criminal proceedings thus functions as a critical juncture that temporarily narrows communicative latitude, before corporations gradually reassert control over the narrative in subsequent communications.

## Discussion and conclusions

Our results show that companies do more than simply inform the public or stakeholders about ongoing cases, and that they go beyond attempting to repair their reputation. Corporations communicate in response to legal, social, and economic constraints and incentives — and when they do, they actively participate in shaping the public understanding of their own criminal conduct. Read through the lens of Tombs and Whyte’s (2015) decriminalization framework and Edelman’s (2016) concept of symbolic compliance, corporate communication during criminal proceedings emerges not as a mechanism of transparency but as a mechanism for managing transparency: for satisfying the minimum requirements of institutional visibility while systematically avoiding the deeper acknowledgment that genuine accountability would require.

A key finding of this study is that corporate communication about corruption is overwhelmingly reactive rather than voluntary. Communication patterns and timing are shaped by various factors, including company structure, procedural decisions in Switzerland or abroad, and the intensity of media or public pressure. It supports Blanc et al.’s (2019) findings about external pressure driving corporate communication, while extending that observation to show that even pressure-driven communication remains strategically managed. This corporate silence also resonates with Tombs and Whyte’s (2015) argument that decriminalization is the path of least resistance for corporate offenders: not communicating preserves the ambiguity that structured irresponsibility produces, while communicating incurs both reputational and legal costs.

Another contribution of this study is the identification of four distinct frames through which corporations present their own corruption cases: criminal, illegal, managerial, and righteous frames. Each serves a specific function, reflecting different levels of acknowledgment, responsibility, and reputation management. Moreover, each can be understood as a discursive instrument of decriminalization in Tombs and Whyte's (2015) sense.

The managerial and illegal frames are the most direct expressions of this logic. The managerial frame removes misconduct from legal discourse altogether and recasts it as a technical error or strategic misstep stripping it of both criminal and moral connotation. The illegal frame acknowledges wrongdoing but systematically avoids criminal law terminology. Both frames reproduce at the level of language what Tombs and Whyte (2015) identify as structured irresponsibility at the organizational level: the diffusion of accountability through the deliberate erosion of the criminal register. The illegal and managerial frames are, in this sense, good examples of how corruption can be portrayed as the mistakes of a few individuals, thereby downplaying its systemic significance and stabilizing economic legitimacy (Hess & Ford, 2008).

The righteous frame, which explicitly denies wrongdoing and asserts the legitimacy of corporate conduct, represents the most confrontational form of resistance to criminalization, deployed characteristically in the early stages of proceedings and under acute public pressure. As our findings show, it is invariably abandoned once criminal justice authorities make their conclusions public, illustrating both the limits of this posture and the degree to which judicial authority, when exercised publicly, constitutes a constraint that even powerful corporations cannot override indefinitely.

The criminal frame, and particularly its consensual variant, occupies a more ambivalent position. On its face, this frame appears to represent accountability: corporations acknowledge their conduct in criminal law terms, refer to prosecuting authorities correctly, and describe proceedings as what they legally are. Yet read through Edelman’s (2016) framework, this apparent transparency performs compliance while simultaneously reframing criminal proceedings as collaborative achievements. Statements emphasizing voluntary cooperation, proactive settlement, and compliance reform construct an image of the corporation as a responsible institutional actor rather than a criminal offender. This can be understood as symbolic compliance: the language of criminal responsibility is adopted in a way that ultimately serves to normalize and contain the criminal episode rather than to open it to systemic critique.

Perhaps the most theoretically significant finding of this study is the sequential logic of framing across the stages of criminal proceedings. Companies initially seek to minimize or deny the severity of corruption until such a narrative is no longer credible. They adopt the criminal frame at the moment of NTR conclusion before progressively reintroducing illegal and managerial frames in subsequent communications. This dynamic is not random; it follows a clear institutional logic that extends Edelman’s (2016) framework in an important direction: symbolic compliance is not merely structural but also temporal. Corporations perform criminal acknowledgment at the precise moment when it is institutionally unavoidable, then strategically withdraw from it as institutional pressure subsides.

This sequential pattern also reveals that corporate narrative management is reactive and contingent rather than pre-designed. Corporations adapt their framing strategies to each new development — judicial decisions, media coverage, foreign proceedings — suggesting that the communicative work of decriminalization is an ongoing negotiation with an evolving institutional environment, rather than a coherent strategy deployed from the outset. The framing of corporate corruption is therefore not merely a linguistic choice, but a powerful and adaptive tool of reputational management that shapes how misconduct is perceived, remembered, and ultimately judged beyond the criminal justice system.

A further finding of this study is that even when companies refer to their corruption cases, their communications often remain superficial and at times cryptic. Readers receive minimal substantive information and can sometimes only infer that a company is reporting on a criminal case at all. This is consistent with synoptic dimension of corporate power: the active management of corporate self-visibility in ways that naturalize institutional legitimacy. This narrow communication strategy, designed to satisfy investors and direct stakeholders rather than the broader public, undermines the wider social value of transparency.

In sum, this study extends criminological understanding beyond traditional victim–offender–justice dynamics by examining how powerful actors construct self-serving narratives to manage stakeholder expectations during criminal proceedings. It contributes to the criminology of corporate crime by documenting the discursive mechanisms through which decriminalization and symbolic compliance operate in practice, as seen through the corporations’ own communications at a moment of acute institutional pressure.

Future research should examine whether corporate communication differs in cases pursued through ordinary public criminal proceedings, such as the recent trial of Trafigura in Switzerland, where corporations exercise less control over the flow of information and where the sustained public visibility of a trial may alter the temporal dynamics of framing identified here. Comparative research across jurisdictions and industries would further illuminate how institutional context shapes the repertoire of discursive strategies available to corporate offenders, and how different regulatory environments constrain or enable the narrative management of corporate crime.

## Footnotes

1 ESG stands for Environmental, Social, and Governance**.** It is a framework used to assess how companies manage risks and responsibilities related to the environment, their social impact, and their internal governance practices.

2 For a recent literature review of anticorruption disclosure practices, see Khelil, Khlif, and Achek (2024).

3 A criminal proceeding is the formal process by which the state investigates, prosecutes, and sanctions a person or entity for conduct defined as a criminal offence (see Swiss Criminal Procedure Code of 5 October 2007, CPC, SR 312.0).

4 Summary Penalty Orders are sometimes entitled as Summary Punishment Order, see, for example, the report of the OECD Working Group on Bribery from 2019, p. 27. Other prominent NTR instruments include the Plea Agreement (PA) in the United States, likewise a conviction-based mechanism. Prominent non-conviction-based instruments are the Deferred Prosecution Agreement (DPA) in the United States and the United Kingdom as well as the Convention judiciaire d’intérêt public (CJIP) in France (OECD 2019).

5 In the context of criminal proceedings involving transnational corruption, the OAG is the counterpart to the British Serious Fraud Office (SFO), the French Parquet National Financier (PNF), and the American Department of Justice (DoJ).

6 Some corporations also employ sustainability or corporate social responsibility (CSR) reports. An initial analysis revealed, however, that these instruments did not contain communications substantively different from those in the annual reports. This study therefore relies on press releases and annual reports as the most consistently used instruments across all corporations.

7 While the starting point for data collection were the English-language websites of the corporations, material in English, French, and German was analyzed.

8 Some corporations face prosecution in multiple countries — for example, SBM Offshore in the Netherlands, the United States, and Brazil, or ABB in South Africa and the United States.

9 For an overview of the Odebrecht case, see, for example, Castro and Crosta (2020).

10 For instance, policies such as the EU Market Abuse Regulations Art. 17, the USA Security Exchange Commission, or national stock exchange guidelines such as SIX Swiss Exchange Art. 53.

11 Throughout the results, most quotations were originally in English; quotations from French or German sources, however, were translated by the authors.

12 See, for example, the *Wall Street Journal*’s article from 13th September 2008, “Swiss Prosecutors Widen Probe of Alstom Payments. Alleged Bribes May Top $500 Million, Investigators Say”; https://www.wsj.com/articles/SB122126539807730749 (last visit, 3.9.2025).

13 Although this translation is technically inaccurate as the official English designation is *Office of the Attorney General of Switzerland.*

14 Kusile is the name of a coal-fired power plant in South Africa, where ABB employees bribed officials of the state-owned utility Eskom in connection with the awarding of contracts.
